# Evaluating True Barriers to Dental Care for Patients with Periodontal Disease

**DOI:** 10.16966/2378-7090.347

**Published:** 2020-12-18

**Authors:** Tina Treloar, Susan S Bishop, Virginia Dodd, Luciana Macchion Shaddox

**Affiliations:** 1 Dental Programs Santa Fe College, Gainesville, FL, USA; 2 College of Dentistry Department of Oral Health Practice, UK Chandler Hospital, Lexington, KY, USA; 3 Department of Health Education and Behavior, Gainesville, FL, USA; 4 Research Division of Periodontology, Center for Oral Health Research, University of Kentucky College of Dentistry, Lexington KY, USA

**Keywords:** Surveys, Dental care delivery, Barriers to dental care, Appointment compliance

## Abstract

**Background:**

The cost of care is often reported as a primary reason why patients fail to seek dental treatment; however, this may not the only component.

**Aims/Objectives:**

To examine an underserved population’s perspective on the importance of dental care and barriers they face to seek treatment. The effectiveness of compliance incentives, such as gift cards, was also examined.

**Methods:**

We conducted a survey study to gain insight of an underserved population’s perspective of barriers to care and correlated the reported barriers with the true reasons of missed appointments in our study cohort. Appointment compliance was also examined before and after implementation of gift card incentives, appoitment reminder letters and additional staff.

**Results:**

Most patients felt dental care was important/very important to them. However, no correlations were found between survey responses and true reasons for missing appointments. Eighty-seven percent of patients report having some form of financial difficulty and compliance statistically improved after implementation of financial compensation in this population (69% completed appointments before gift card implementation versus 75% after gift card implementation, p=0.01), but not after the implementation of additional appointment reminders and contact staff.

**Discussion:**

Although the majority of patients reported dental care was important to them, there was an average of 31% missed appointments for patients who completed the survey and no correlations were found between the information patients reported and true reasons for non-compliance. Interestingly, even though care was free of charge, most patients reported to be in some form of financial difficulty and compliance was slightly improved through the implementation of financial compensation. Other potential barriers that need to be further examined include economic barriers, transportation, insurance utilization, and patient anxiety towards dental care.

**Conclusion:**

A survey is a resource to identify reasons why patients abstain from seeking care but may be not the best one as reasons reported do not correlate with true reasons of appointment failure. Financial compensation was shown to improve compliance with appointments. Further information gathering is necessary to gain insight into true barriers to dental care within an underserved population.

## Background

Americans present for preventive dental care most often when they have monetary resources and when they have dental insurance [1]. Age, race, gender and socioeconomic status play a role in dental care utilization. Hispanics and non-Hispanic blacks are less likely to utilize dental services than other races [1]. Additionally, those with private dental insurance had more dental visits than individuals with public or no dental insurance [1]. It is essential to learn more on how to improve and effectively deliver care by assessing patient’s perceptions to needs and barriers.

In a study of unmet health care services, dental care was a more common unmet health need than other types of health services [2]. New methods are needed to learn more and implement change to better increase dental care utilization among children, especially among some ethnic groups. For instance, African American children are twice as likely as white children to have substandard health condition and dental status [3]. These authors also noted that African American children were less likely to access dental care for a preventative dental appointment than other racial and ethnic groups [3]. Thus, it is important to also recognize the prevalence of oral disease in this ethnic group. Localized Aggressive Periodontitis (LAP) is an aggressive but less common form of gum disease that affects younger individuals. African Americans have a greater prevalence than those in other ethnic groups of having aggressive periodontitis [4]. LAP affects specific teeth in the mouth, first molars and incisors, with severe and rapid bone loss, and patients are usually found to be systemically healthy [5]. Thus, routine dental care is the first step in diagnosing this condition. If dental visits are not occurring, then Initial diagnosis is likely to be missed, and the disease is likely to progress rapidly leading to the loss of affected teeth at a young age.

Financial hardship is documented as a primary barrier to dental care. According to Vujicic M, et al. regardless of age, amount of revenue, and source of insurance, greater numbers of people described economic hurdles for dental care, compared to other forms of health services [6]. African Americans with less education, lower income, and more children described poor oral health and documented reduced utilization of dental services [7]. Other potential barriers include: economic barriers, transportation, public insurance utilization concerns, and anxiety [8].

A unique opportunity became available to evaluate barriers to care during an existing NIH funded clinical study (Clinical Trial registration: #NCT01330719 at clinicaltrials.gov) evaluating children diagnosed with Localized Aggressive Periodontitis (LAP) and periodontally healthy patients. This opportunity was to further investigate why patients of a similar racial background and economic status miss dental appointments, even when there is no cost associated with the visit.

## Methods

### Inclusion criteria and population

For our clinical study population, patients within this study were enrolled who were ages 5–25 and were recruited from approved clinic locations where patients are treated: at the University of Florida, Gainesville, Florida, as well as various Florida health departments, and other clinic locations, which include Leon, Duval, Jackson and Collier counties. A survey questionnaire was utilized to assess the obstacles that may prevent patients from seeking dental care in order to identify and understand the barriers to care and to help develop a better strategy to improve appointment compliance within this population.

In order to be eligible for the survey, study patients were to be enrolled in the clinical study or have a child or children enrolled in the clinical study. Patients must also have indicated on the clinical ICF (informed consent form) from the study that they wished to be contacted for future studies. Failure to meet these inclusion criteria would exclude them from participating in the present survey study. Qualifying patients were approached by a research staff member at the time of/their own or their child’s dental appointment in the study clinic site. They were then asked if they were interested in participating in a study evaluating barriers to dental care. If they agreed, a research staff member described the purpose of the study and what would be asked of them, specifically that the study involved answering a short questionnaire investigating potential barriers that may prevent patients withdraw their consent to participate at any time without any impact on their own or their child’s involvement in this or any other research study. Upon review of the responses to the questionnaire, a research staff member could seek verbal clarification and/or elaboration of certain answers when needed. Research staff also had the option to assist the patient/parent in completing the questionnaire if literacy was a barrier for accurate reporting.

### The survey

The survey consisted of 19 questions- multiple choice and open-ended questions. Questions were presented which assessed the amount of effort required for patients to come to dental appointments. Factors considered for this were the patient’s perception on the amount of effort for travel, the time and distance in miles the patients must travel their mode of transportation and how likely they would miss an appointment due to their mode of transportation’s reliability or transportation related financial issues. Patients were also asked about their perception on the importance of care and their anticipated outcome of the treatment. Additional barriers were assessed which would keep patients from their appointments such as lack of childcare, school and work commitments, and any other obligations they would like to include in an open-ended portion of the survey. Patients were also asked to provide feedback on how dental appointments could be made less stressful for them and if they had any additional recommendations regarding their dental appointments. Finally, patients were asked to share information regarding their financial situation.

### Correlation with missed appointments

To determine a generalization of true appointment compliance during the clinical trial, subset from the ongoing clinical trial, from which our study population was generated. We evaluated 10 years of data between the years of 2006–2016 which included patients with LAP, healthy patients, and siblings of diseased patients. Appointment compliance rates were reviewed to determine the effectiveness of implementation strategies during the clinical trial such as increased number of clinical staff, appointment letter reminders, and gift card incentives. Appointment compliance was assessed and categorized based on missed versus completed appointments. A total of 1,578 appointments were evaluated and categorized as either completed or missed. Appointments were considered completed if a patient arrived and stayed to the end of the appointment. Appointments were considered missed if the patient gave less than 24 hours’ notice when cancelling, or if the patient did not show up for the appointment. Once we assessed the total of missed verses completed appointments, we investigated further details regarding these missed appointments before and after implementations: reminder letters, increased staff, and gift cards.

### Reminder letters

In addition to an existing protocol to remind patients of their appointments with phone calls, we added reminder letters and mailed these to patients approximately two weeks prior to scheduled appointments.

### Gift cards

Initially this study did not include gift cards. After some time into the study we got approval to dispense these as a way to improve compliance with appointments since this was a long study with several follow ups. Gift cards were given upon completed appointments. Patients could receive up to $60 in Visa gift cards, dependent upon the study length option and compliance. After patients were deemed qualified, consented and completed the baseline protocol, a $10 gift card was distributed directly to the patient at the end of the first (baseline) appointment. Patients would receive an additional $25 gift card if they successfully completed two consecutive maintenance appointments within the study protocol criteria and timeline. Upon successful completion of the study (24 months), patients would receive a $25 gift card at the final appointment.

To ensure security and validity of the gift cards, all gift cards were distributed directly to and activated by the patient. A signature and date was recorded by both the study member distributing the gift card and the patient to acknowledge distribution and receipt of the gift card. A tracking log was utilized by record distribution and signatures. The tracking log and gift cards were housed in a locked cabinet.

### Additional staff

The addition of a dental hygienist was added to the study team on August 1, 2014. The purpose of additional staff was to increase patient interaction during the appointment and increase communication pre-appointment to aid in study protocol and timeline compliance.

### Ambidirectional analysis

In order to determine the effectiveness of appointment compliance motivators and incentives, we performed an am bidirectional analysis of appointment compliance before and after implementation of increased staff, appointment letter reminders, and gift card incentives.

Reported reasons for missed appointments were also investigated for comparison. The reported reasons were given by the patient for their missed appointment when communicating with study staff. If a patient was unable to be contacted, the study staff would list ‘unable to contact’ and ‘no show’ to categorize the missed appointment. The true reasons were then compared versus what had been reported as possible barriers on the survey by the same patient/family member. A section was included in the survey which asked an open-ended question for patients to report reasons that they anticipate missing an appointment in the future. The cross comparison of potential reasons for missed appointments reported on survey by patients with actual missed appointment reasons documented by study staff was evaluated only for the patients/families who completed the survey.

### Statistical approach

We performed analytical comparison before and after incentives using the Chi-Square test with Yates’ correction using the Graph Pad Prism software version 9.0(Graph Pad, San Diego, CA).

## Results

### Survey

#### Importance of dental care:

Figure 1A shows that 82% of respondents to the survey rate that dental care is very important to them while 18% report it is important. One hundred percent of the respondents indicated that dental care was very important or important to them versus other choices.

#### Distance and Transportation to dental center:

Regarding transportation and travel, 62% of patients reported no effort to get to their dental appointment, while 31% reported “not much” effort, 4% reported “a little effort” and 2% reported “a lot” of effort (Figure 1B). Most (62%) reported living less than 10 miles of their dental clinic location. Travel time to dental appointments is under ten minutes for 9% of the patients, 10–19 minutes for 31%, 20–29 minutes for 16%, 30–30 minutes for 24%, 40–49 minutes for 9%, over 50 minutes for 9% and 2% did not report on the time. In regard to missing an appointment due to the length of driving time to the clinic, the following was reported: 71% reported it would be “Very unlikely” to miss, 27% reported “Unlikely” and 2% “Likely”. When asked how likely it would be to miss a dental appointment due to heavy traffic, 53% said it would be “Very unlikely”, 36% reported “Unlikely” and 11% “Likely”. For means of transportation, 78% of patients travel in their own car to appointments, 2% use someone else’s car, 7% get a ride from someone else, 7% take the bus, 2% walk and 4% work within the building location. 54% of participants reported it is “Very unlikely” they would miss an appointment due to car trouble, 42% reported it was “Unlikely” and 4% said it would be “Likely”. Sixty percent of participants reported it would be “Very unlikely” they would miss an appointment because they could not get a ride, 33% said it would be “Unlikely” and 7% said it would be “Likely”.

#### Other Barriers: work, childcare, school, medical care:

Regarding additional barriers outside of transportation, 47% of participants reported it would be “Very unlikely” to miss an appointment due to work obligations, 42% report it would be “Unlikely”, 4% said it would be “Likely” and 7% said it would be “Very likely”. For appointments missed due to school, 53% it would be “Very unlikely”, 36% reported “Unlikely”, 7% reported “Likely” and 4% reported “Very likely”. 76% said it would be “Very unlikely” to miss an appointment due to lack of a babysitter, 22% reported “Unlikely” and 2% “Very likely”. Participants were also asked open ended questions including, “Are there other reasons you might miss a dental appointment?” 76% reported “No”, 2% reported “Death in the family”, 4% “Emergency”, 2% reported “School”, and 14% reported “Sick” and 2% “Weather (Figure 2A).

#### Financial issues:

Questions concerning financial issues which may keep patients from their dental appointments were also evaluated. Assessment revealed that 60% of patients reported it would be “Very unlikely” to miss an appointment due to lack of money for gas, 33% said it would be “Unlikely” and 7% said it would be “Likely”. Similarly, participants were also asked how likely it would be to miss an appointment due to lack of money for the bus. 76% stated it would be “Very unlikely”, 20% reported “Unlikely” and 4% “Likely”. Participants were then asked to select “Which of these statements best describe your present financial status?” 2% selected “Money is not a problem. I can buy about whatever I want”, 27% selected “I have enough to manage plus some extra”, 63% selected “I manage to get by”, and 8% selected “I do not want to answer this question”. Finally, patients were asked “If you were faced with an unexpected $500 medical bill that was not covered by insurance, how would you best describe your situation?” 9% reported “I am able to pay the bill without any problem.”, 42% stated “I am able to pay the bill but it will be hard.”, 45% stated “I am not able to pay the bill.” and 4% elected to not respond to the question (Figure 2B).

#### Patienťs perceptions of dental appointments:

Patient’s perceptions of the appointments were investigated. After completing the series of dental appointments, 85% of the survey participants felt either their mouth or their child’s mouth would be “A lot more healthy”, 11% thought it would be “A little bit healthier” and 4% reported they were not sure. 82% of participants felt it was “Very important” to come to every dental appointment and 18% felt it was “Important” (Figure 1A). Participants were then asked an open ended question of “How can we help make your dental appointments less stressful for you?” 78% reported “Nothing”, 2% requested a “Gas voucher”, 2% requested “More information before appointments”, 4% requested “More reminders”, 12% requested “More appointment times” and 2% requested “Less discomfort” (Figure 3A).

#### Reasons for non-compliance with appointments:

The reasons reported for missed appointments from the survey respondents and the times for actual missed appointments by these respondents were then evaluated. Among the 79 individuals who responded from the survey, there were a total of 483 appointments assessed within the range of their participation in the clinical study, from 2010–2015, and at the time of the survey study. Of the 483 scheduled appointments, 110 were missed for an average of 23% non-compliance. A breakdown of reported reasons for the missed appointments is a follows: 20% due to carelessness, >1% due to a death in the family, 9% due to illness, 6% had a scheduling conflict on their end, 3% due to transportation and 4% due to weather. There were a total of 64% unreported reasons because the patient did not call or come to the appointment and was not able to be reached (Figure 3B). No correlations were found between the reported survey results and the actual reasons patients missed appointments.

#### Reminders, increased staff, and gift card implementation:

Further assessment was completed for additional appointment factors such as letters to remind patients of upcoming appointments, increased staff, and implementation of gift cards. Prior to the implementation of the reminder letters, a total of 1,536 appointments were assessed from August 2006 to mid-April 2014 with 31% missed (476) and 69% completed (1060). Appointment compliance was assessed from late April 2014 to October 2014after letter reminders with a total of 40% missed appointments (293) and 60% completed appointments (437) (Figure 4A). No statistical difference was found on compliance rates before and after letter implementation (Chi-Square test with Yates’ correction p=0.750).

Additional staff for increased patient interaction was also implemented. Appointments were assessed for all patients from August 2006 to May 2016. Additional staff of one dental hygienist was implemented on 8/1/2014. Prior to the additional staff, a total of 1,545 appointments were evaluated. A total of 29% of appointments were missed (457) while 71% (1,101) appointments were completed. After implementation of additional staff, a total of 567 appointments were assessed. Thirty-two percent (179) of appointments were missed while 68% (388) were completed (Figure 4B) but no statistical difference was found (Chi-Square test with Yates’ correction p=0.172).

**Figure 5:**
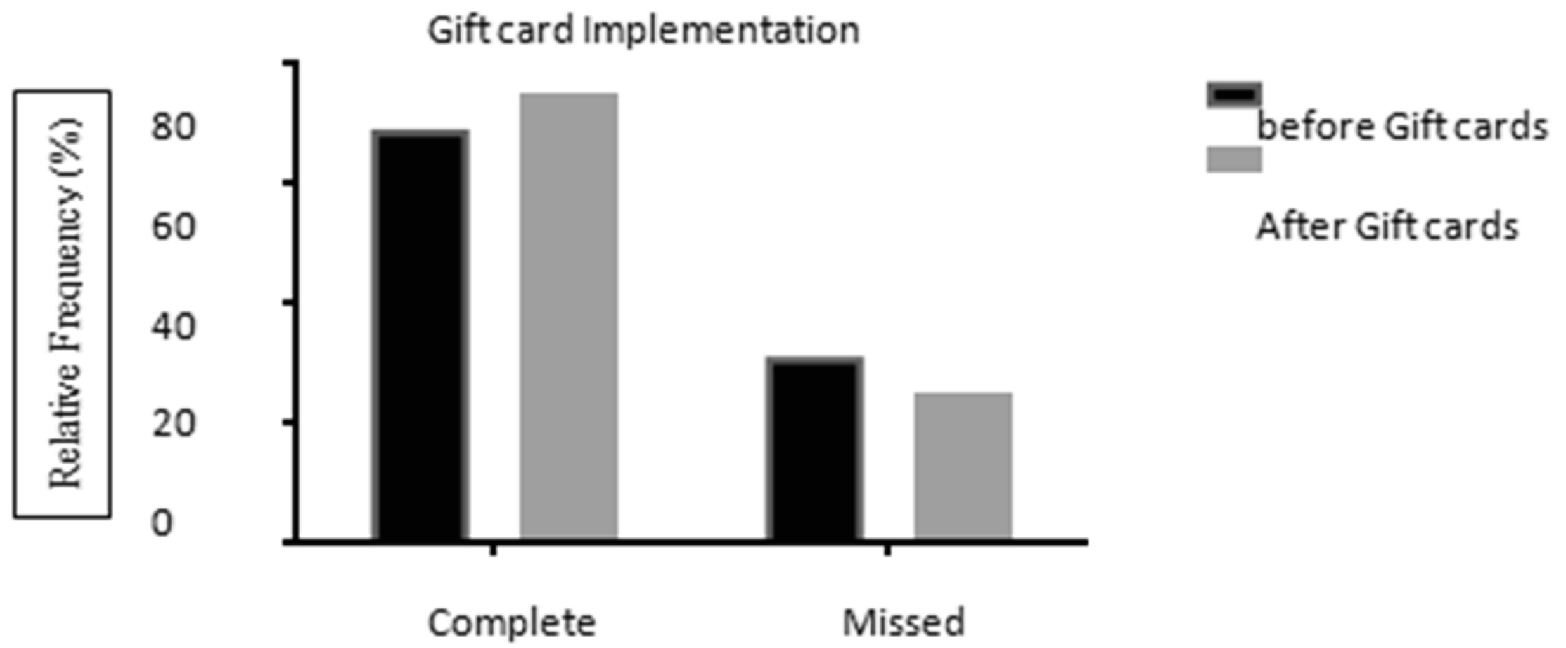
Missed appointments rate after implementation of gift cards lower than before implementation, p=0.0158 by Chi-Square test with Yates’ correction. N (before)=218 missed and 483 completed appointments (701 total). N (after)=159 missed and 477 completed appointments (636 total).

Effect iveness of appointment compliance with the implementation of gift cards was evaluated. All appointments were assessed from January 2011 to October 2016. Gift cards were implemented on April 2016 in which patients could receive up to $60 in Visa gift cards, dependent upon their study length and compliance. Prior to the implementation of gift cards, a total of 701 appointments were evaluated. Thirty-one percent (218) of patients missed their appointment while 69% (483) completed their appointment. After the implementation of gift cards, a total of 636 appointments were assessed. The number of patients who missed an appointment was 25% (159) while 75% (477) completed their appointments (Figure 5). There was a significant difference on completed appointments before and after the incentive was initiated (p=0.0158).

## Discussion

Upon reviewing feedback provided by patients, all requests for assistance in making appointments less stressful for patients were recorded and addressed. No significant changes in appointment compliance were shown through the implementation of increased staff, and increased appointment reminders (Figures 4A and 4B). However, there was a significant better compliance with appointments after gift card implementation (Figure 5). “A dominant void exists in the evidence pool regarding what is successful in recruitment and retention [9]”. Other factors that could impact appointment compliance could be non-quantifiable such as confidence and personal association or familiarity, which increased staff and communications could potentially address. However, the present study only showed effects of gift card implementation, which does agree with reported financial difficulties faced by this underserved population. Additionally, non-quantifiable factors such as cultural acceptance, angst, or a practice of self-medicating could also be attributed to lost appointments but were not evaluated here. For instance, in an African American population located in the Southeastern U.S., evaluation of oral health demands and hurdles revealed that anxiety and entry were the highest reported hurdles to care [10].

Patients largely report dental care as important to them and in fact, literature reports that most Americans do have access to dental care and do seek regular care [11]. However, access to dental care is hard to measure due to the many factors usually involved [12]. In the present study, we still reported average of 31% missed appointments among the patients. No correlations were found between the information patients reported and the true reasons reported for non-compliance with actual appointments. Interestingly, even though care from this study was free of charge, most patients reported having some degree of financial difficulty, and thus this could explain an increase in appointment compliance demonstrated after the implementation of financial compensation with gift cards. In fact, lower percentage of visits to the dental office in the preceding year has been reported for populations below poverty levels when compared to populations at or above these levels [11]. Another study corroborates with our results here, in the evaluation of web-based intervention to decrease obesity risk among minority youth using a variety of strategies, including gift card incentives. The study suggested that gift cards provided to study participants may have led to higher retention rates [13]. This same study concluded that activities for health promotion in a study, specifically those that are aimed to building personal connections and associations are very important for inclusion and retention of Latino and African American families [13]. Trust and relationships were built when study staff developed partnerships with community organizations and when staff attended and tabled at community/school events. These may be important tools especially in more vulnerable populations, such as Hispanics and African-Americans, as these have been reported to have lower access to dental care [12]. Although we evaluated the implementation of an additional staff member and appointment reminders in the present study, those modifications did not result in a significant additional compliance rate.

The present results do not explain why survey participants express value in dental care but still do not come to appointments regularly. This may indicate a questionnaire survey may not be an ideal instrument to evaluate true barriers to care. Future recommendations for research methods could include a focus group from the underserved patient population which would allow for in-depth discussion and insight into potential and true barriers possibly missed by a questionnaire survey alone.

The consideration of using Community Health Advisors may be another strategy to increase retention and adherence for future clinical trials. Community Health Advisors have been used in other studies to recruit members of their community to participate in clinical and behavioral research studies. The Community Health Advisor (CHA) is a volunteer who has received research training to serve as a research partner. “Volunteer CHAs can be effective in improving the retention and adherence of minority and low-income women in clinical trials [14].” This could be further evaluated in future studies dealing with underserved populations such as this. Regardless of the program used in the attempt to increase access to dental care, it is important that essential elements are addressed: the demand for care, the dental work force, the economic environment [15], as well as geographical barriers and cultural differences. We also must keep in mind when developing these programs that dental care unfortunately still presents the highest level of financial barriers compared to other types of health care services [6].

## Conclusion

All patients surveyed reported it is important or very important to come to dental appointments. However, over one-third of the same patients missed dental appointments even though, 94% of these individuals reported it takes little to no effort to get to an appointment. The majority of patients reported having some form of financial difficulty and indeed the implementation of financial compensation did improve appointment compliance in the present study population. However, no relationship was demonstrated between appointment compliance and increased staff or appointment reminders. Additionally, a survey instrument to identify possible barriers to care may not be a reliable source of true reasons why patients abstain from seeking dental care as the present results demonstrated that the reasons for missing appointments reported on the survey did not correlate with the true reasons patients reported when actually missing appointments. Thus, more information is necessary to gain insight to the true barriers to dental care within an underserved population. Future research with modifications in previously used methods is needed to give greater insight as to how we can improve access to dental care in underserved populations.

## Figures and Tables

**Figure 1A:**
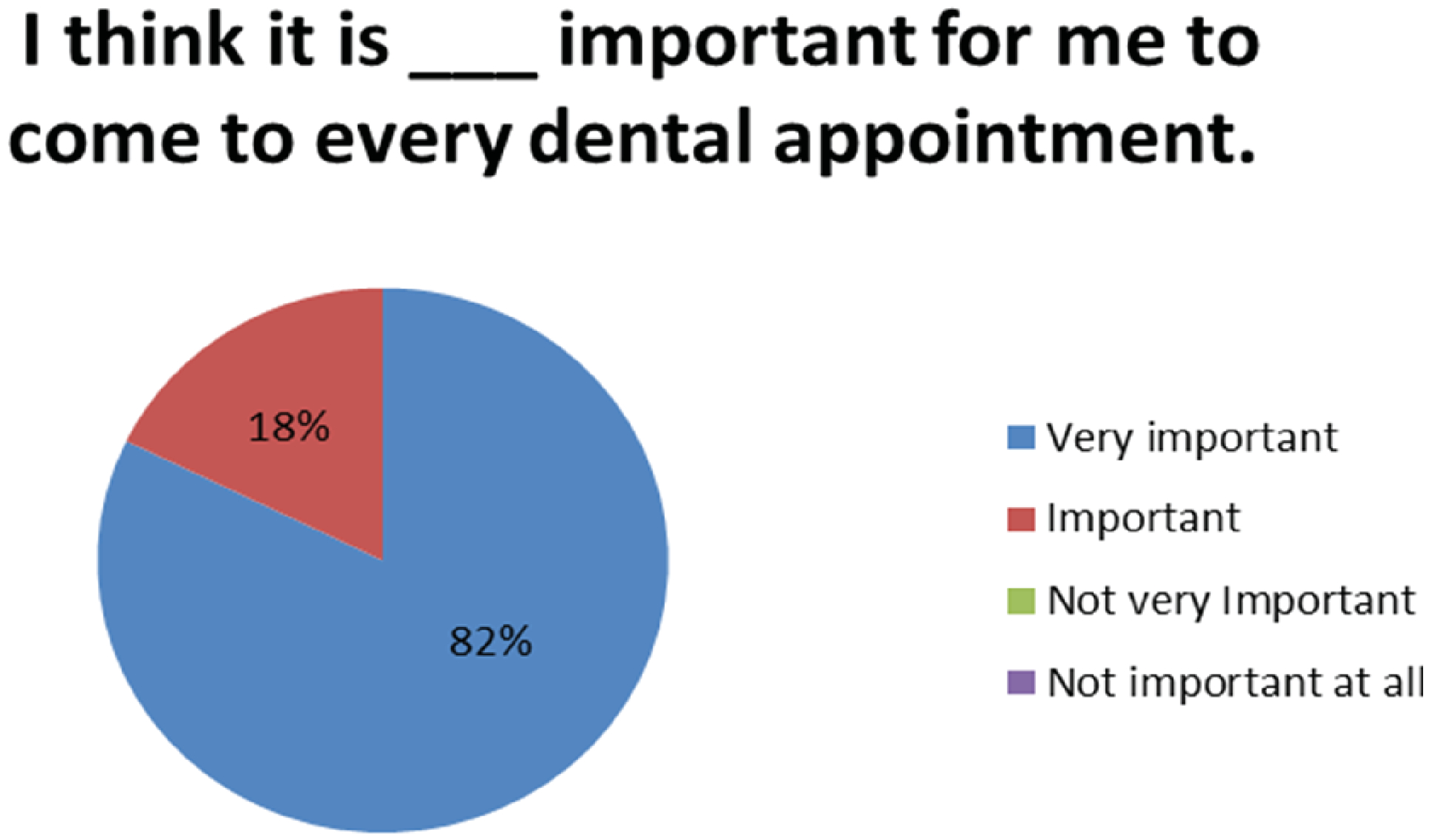
Importance of Dental Care: 100% of patients state it is very important or important to come to every dental appointment.

**Figure 1B:**
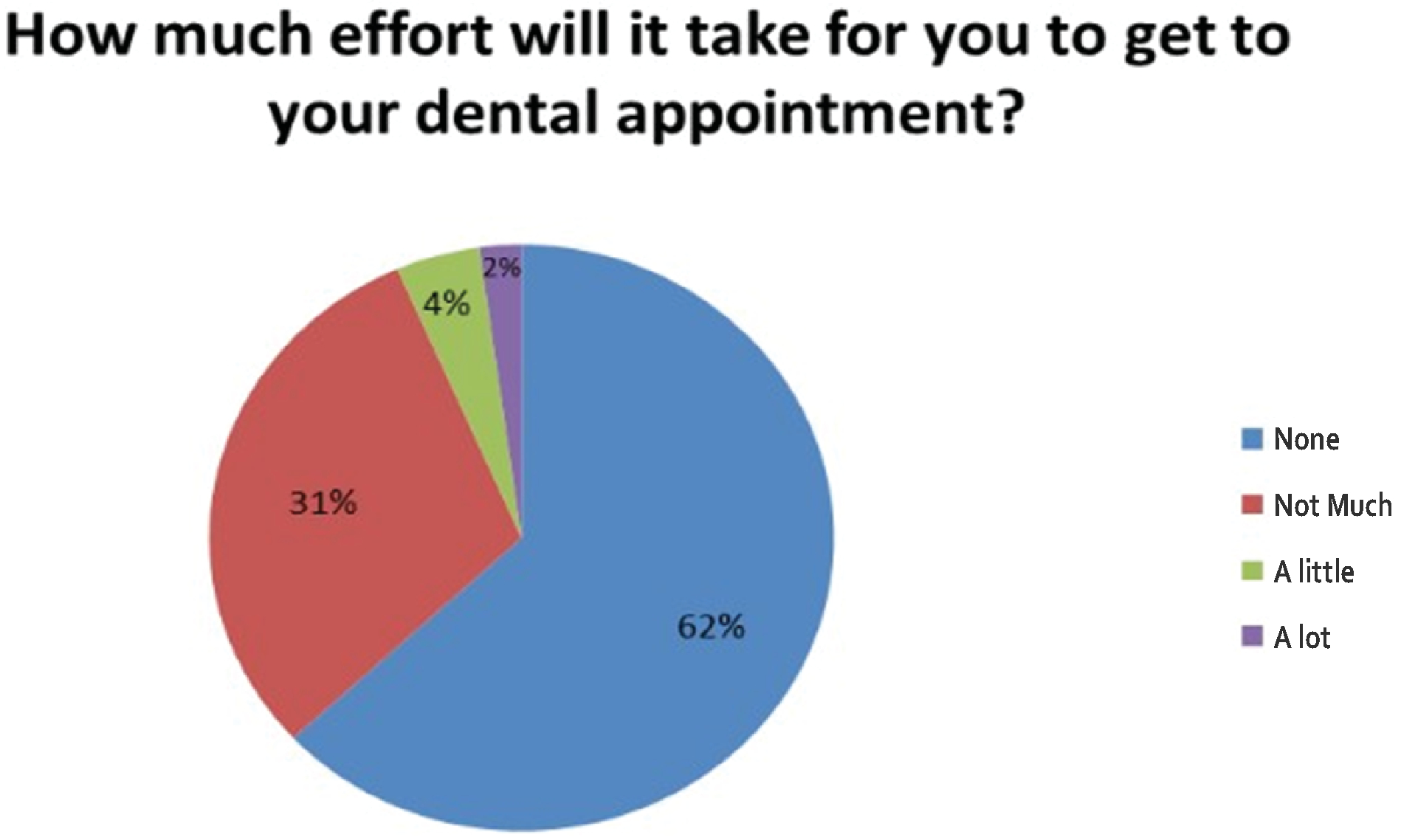
Distance and Travel: Sixty-two percent of respondents mention that no effort is required to get to their dental appointment while 31% report not much effort.

**Figure 2A:**
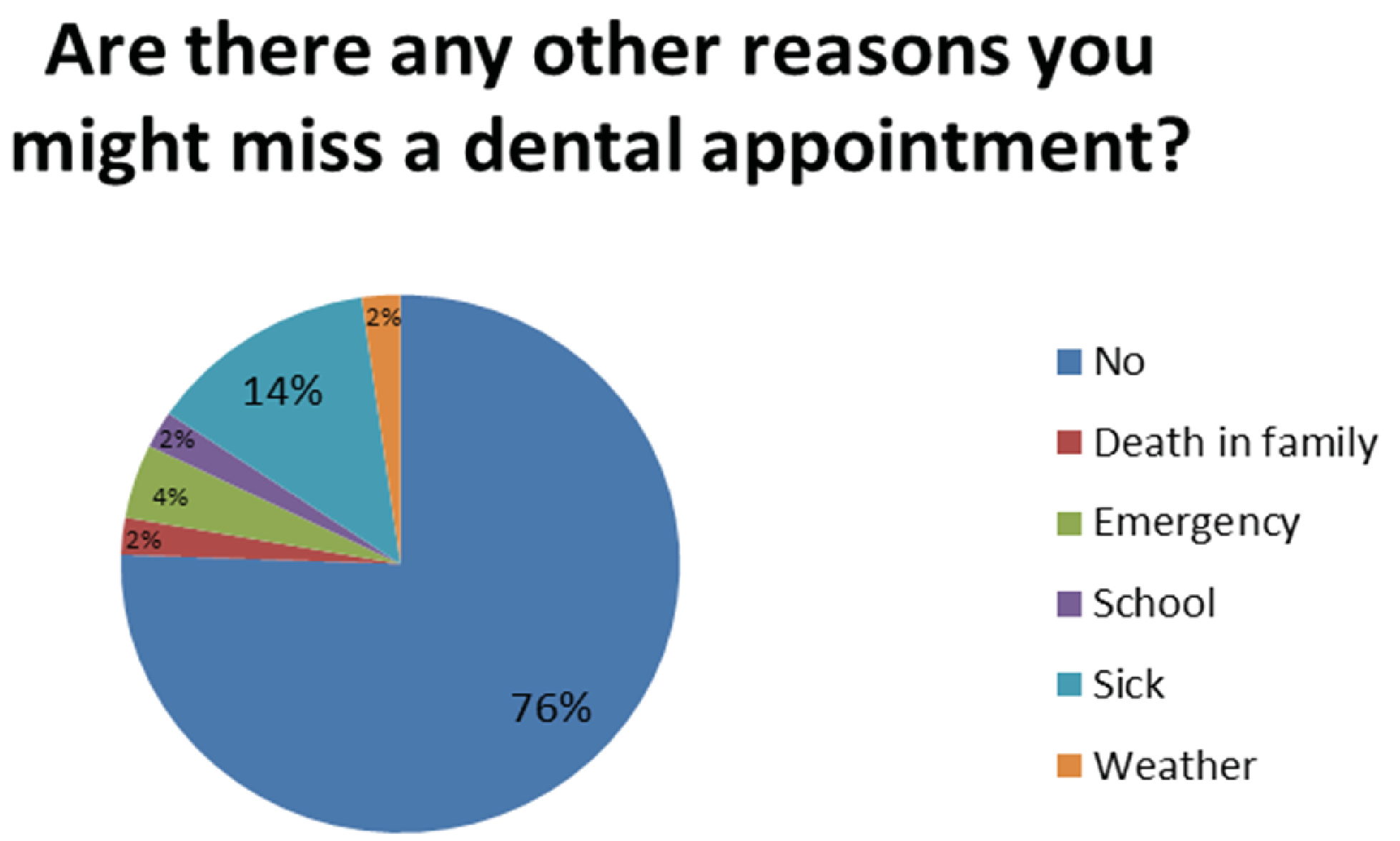
Additional Reasons for Missed Appointments: 76% of patients state that there are no other reasons that they might miss a dental appointment.

**Figure 2B:**
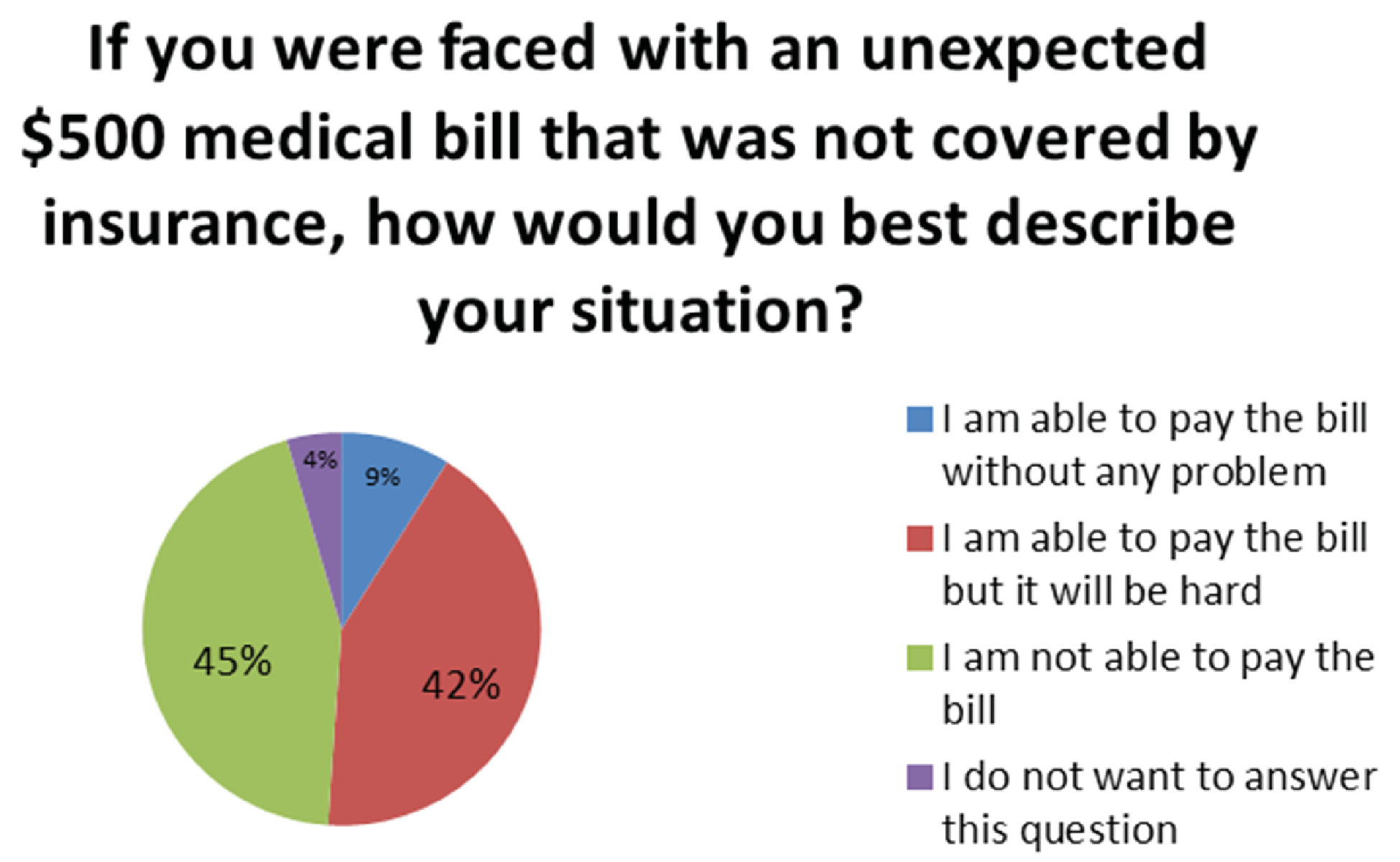
Unexpected medical expense and ability to pay: 87% of patients report they would have difficulty paying for a $500 unexpected medical bill not covered by insurance.

**Figure 3A:**
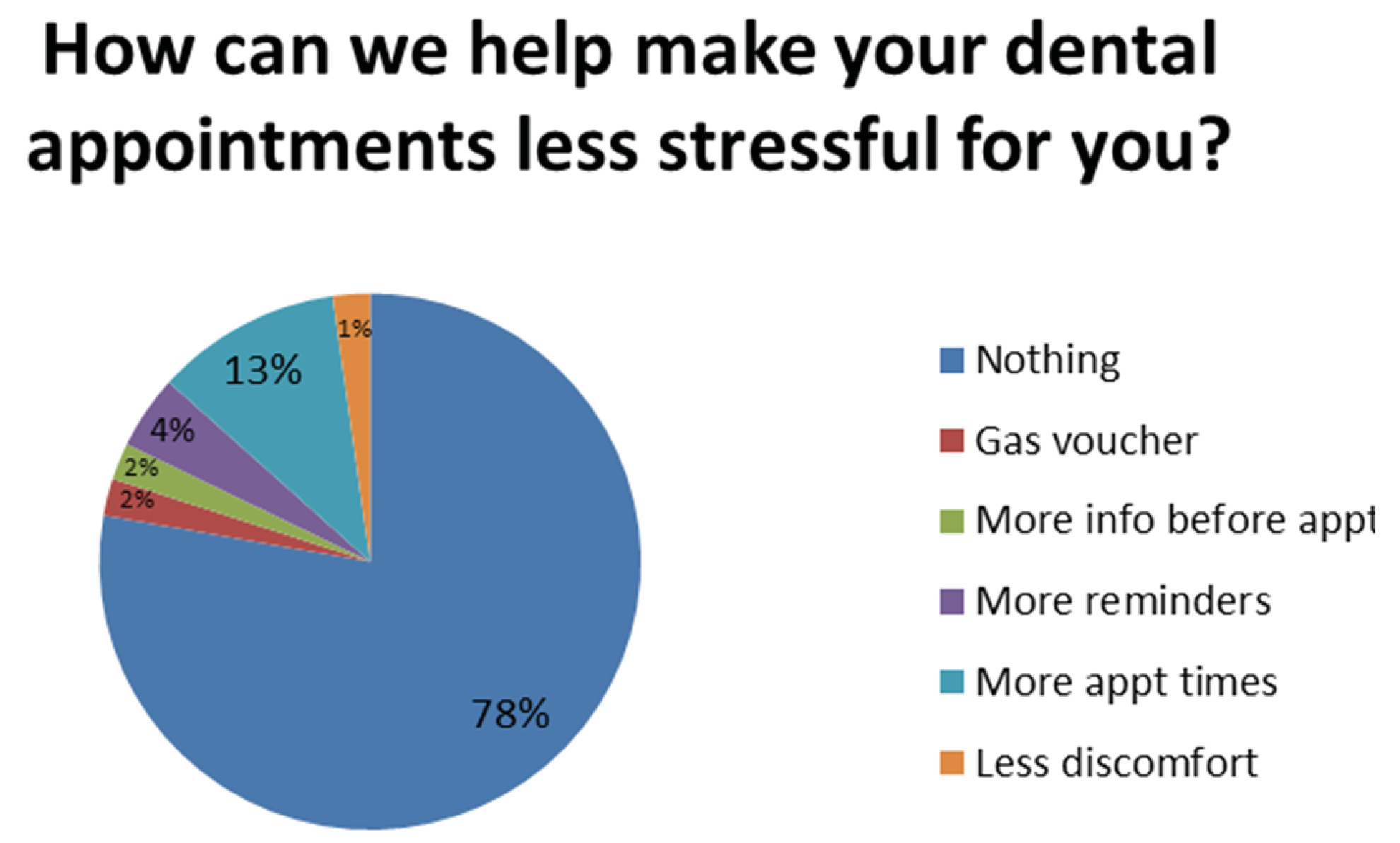
How can we make dental care less stressful: 78% of patients stated that there was nothing that would make their dental appointments (appt) less stressful.

**Figure 3B:**
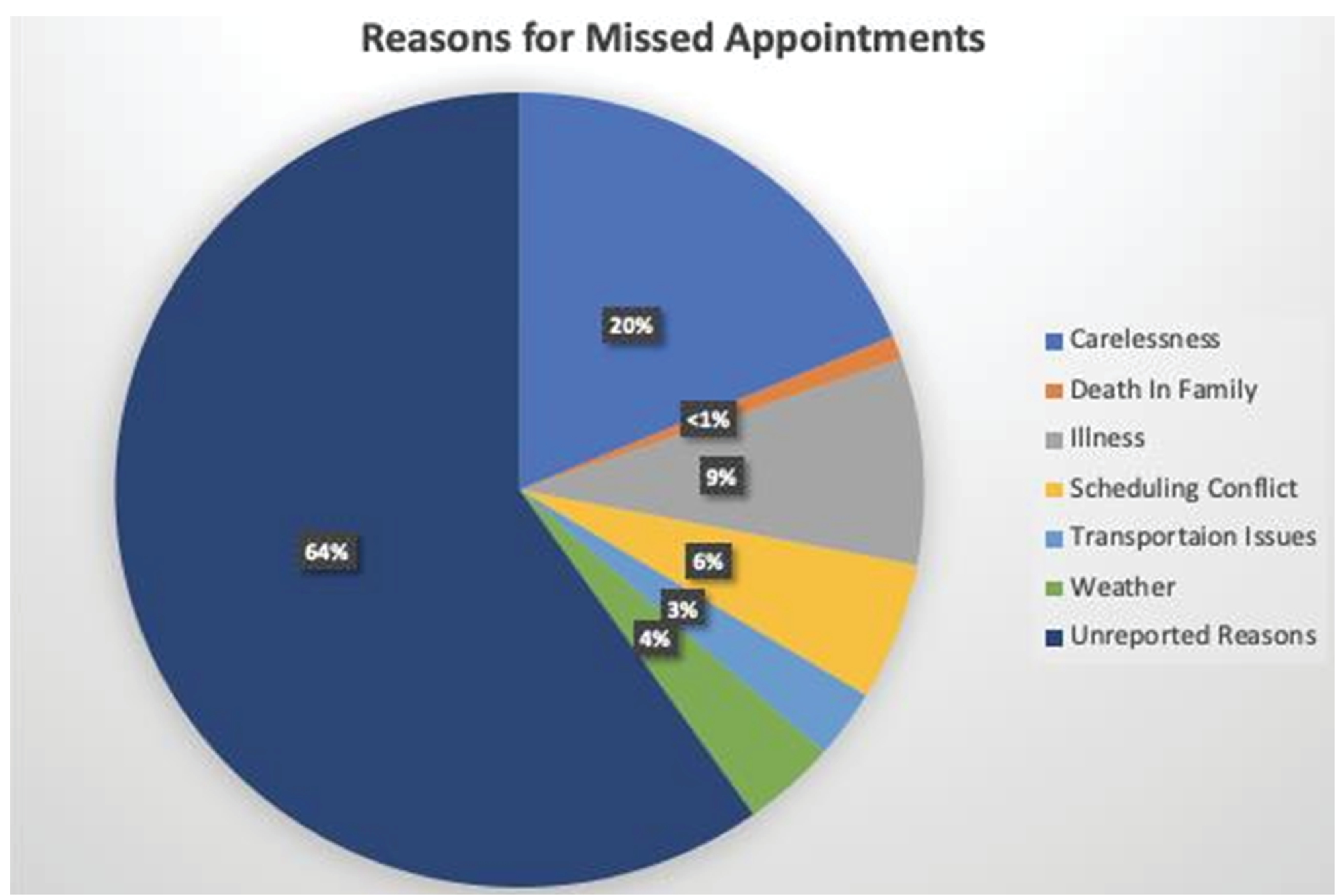
Reasons for non-compliance with missed appointments: 64% of missed appointments were due to unreported reasons. 20% of patient’s missed appointments categorized as carelessness, with “forgetfulness” being the most common reason.

**Figure 4A:**
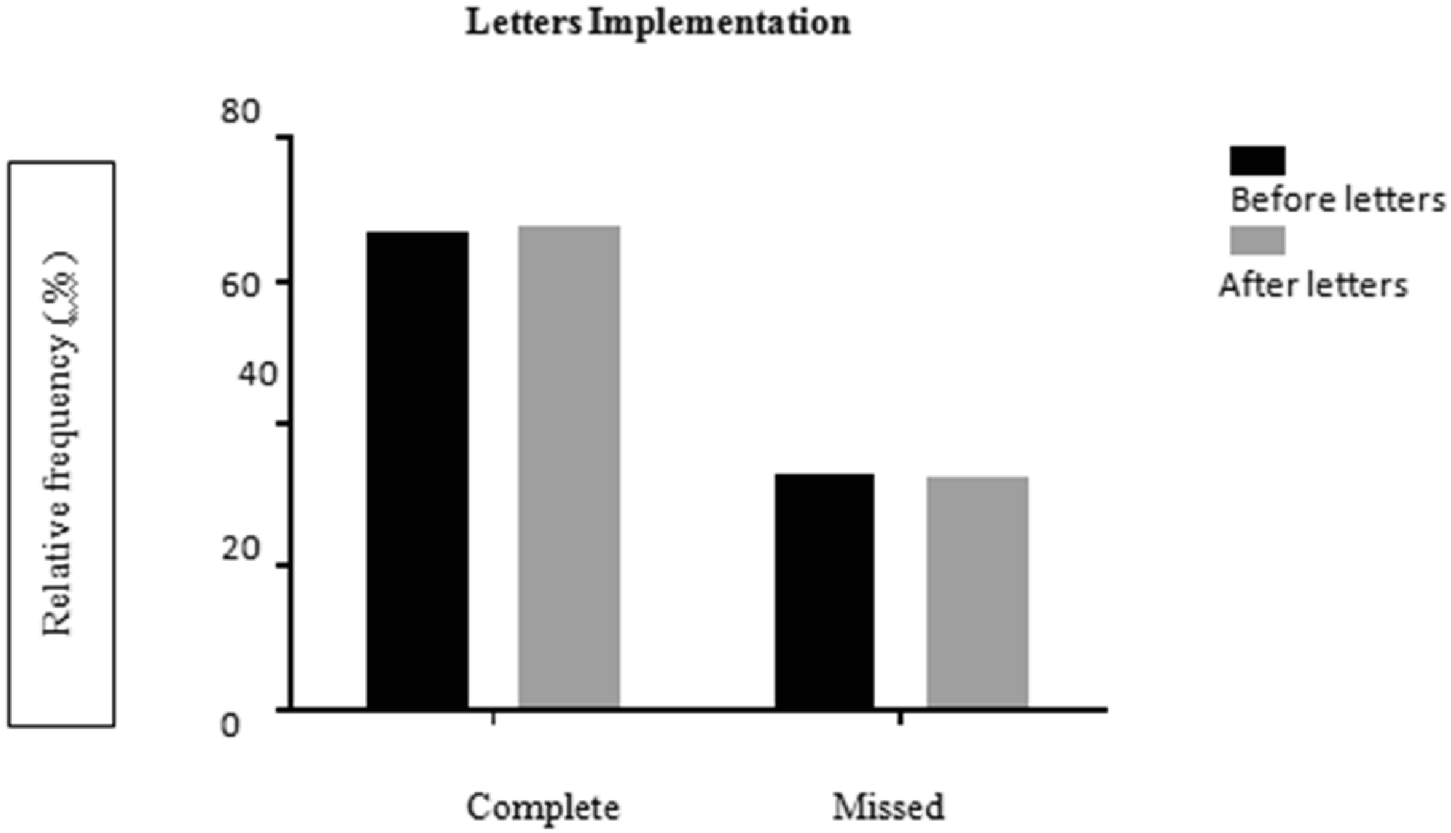
No difference between complete and missed appointment rates before and after implementation of letters of appointment reminders by Chi-Square test with Yates’ correction (p=0.750).

**Figure 4B:**
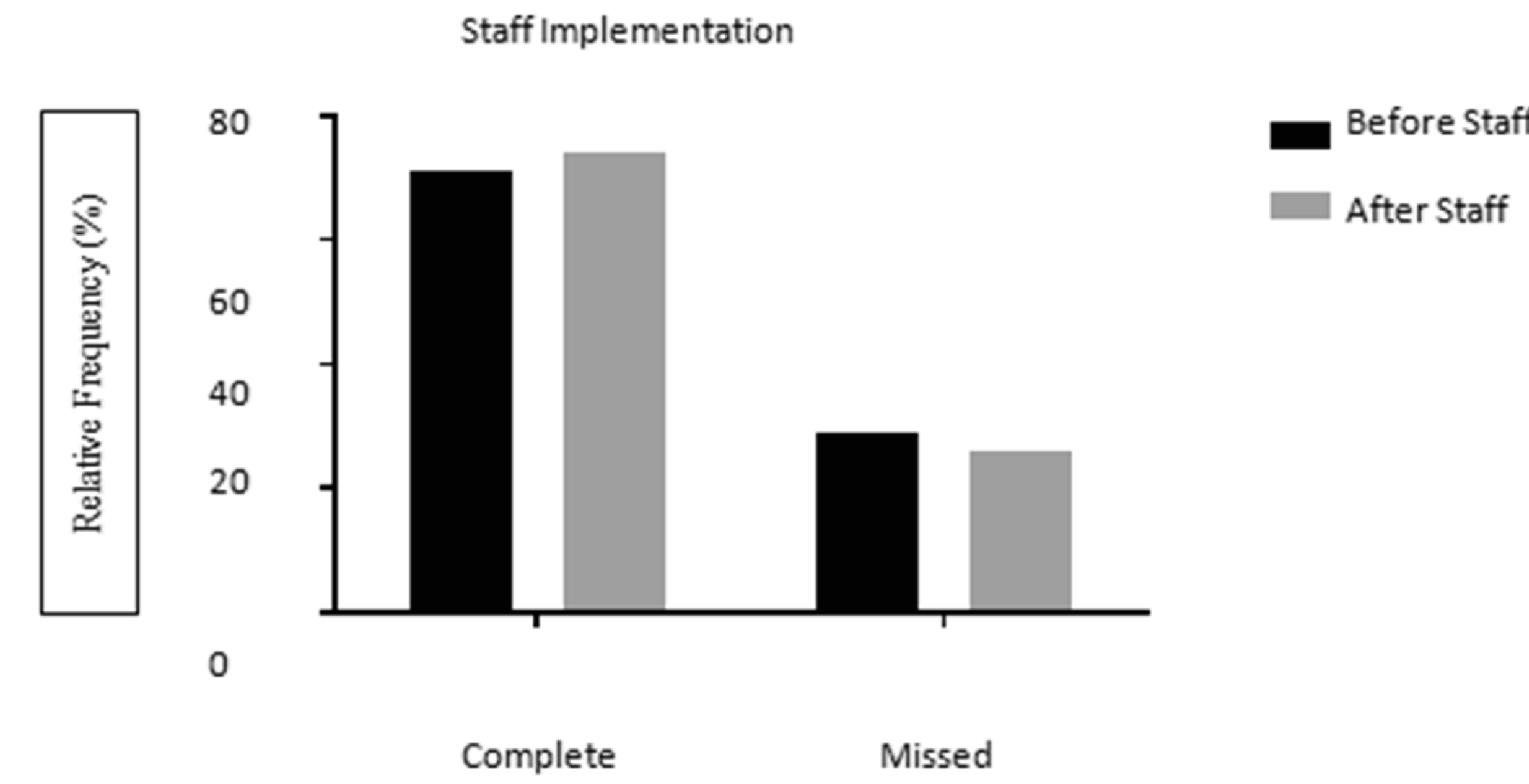
No difference between complete and missed appointment rates before and after implementation of additional staff by Chi-Square test with Yates’ correction (p=0.172).
